# Response Time Threshold for Predicting Outcomes of Patients with Out-of-Hospital Cardiac Arrest

**DOI:** 10.1155/2021/5564885

**Published:** 2021-02-11

**Authors:** Ling Hsuan Huang, Yu-Ni Ho, Ming-Ta Tsai, Wei-Ting Wu, Fu-Jen Cheng

**Affiliations:** ^1^Department of Emergency Medicine, Chang Gung Memorial Hospital, Linkou Medical Center, Taoyuan, Taiwan; ^2^College of Medicine, Chang Gung University, Taoyuan, Taiwan; ^3^Department of Emergency Medicine, Kaohsiung Chang Gung Memorial Hospital, Chang Gung University College of Medicine, Kaohsiung, Taiwan

## Abstract

Ambulance response time is a prognostic factor for out-of-hospital cardiac arrest (OHCA), but the impact of ambulance response time under different situations remains unclear. We evaluated the threshold of ambulance response time for predicting survival to hospital discharge for patients with OHCA. A retrospective observational analysis was conducted using the emergency medical service (EMS) database (January 2015 to December 2019). Prehospital factors, underlying diseases, and OHCA outcomes were assessed. Receiver operating characteristic (ROC) curve analysis with Youden Index was performed to calculate optimal cut-off values for ambulance response time that predicted survival to hospital discharge. In all, 6742 cases of adult OHCA were analyzed. After adjustment for confounding factors, age (odds ratio [OR] = 0.983, 95% confidence interval [CI]: 0.975–0.992, *p* < 0.001), witness (OR = 3.022, 95% CI: 2.014–4.534, *p* < 0.001), public location (OR = 2.797, 95% CI: 2.062–3.793, *p* < 0.001), bystander cardiopulmonary resuscitation (CPR, OR = 1.363, 95% CI: 1.009–1.841, *p*=0.044), EMT-paramedic response (EMT-P, OR = 1.713, 95% CI: 1.282–2.290, *p* < 0.001), and prehospital defibrillation using an automated external defibrillator ([AED] OR = 3.984, 95% CI: 2.920–5.435, *p* < 0.001) were statistically and significantly associated with survival to hospital discharge. The cut-off value was 6.2 min. If the location of OHCA was a public place or bystander CPR was provided, the threshold was prolonged to 7.2 min and 6.3 min, respectively. In the absence of a witness, EMT-P, or AED, the threshold was reduced to 4.2, 5, and 5 min, respectively. The adjusted OR of EMS response time for survival to hospital discharge was 1.217 (per minute shorter, CI: 1.140–1299, *p* < 0.001) and 1.992 (<6.2 min, 95% CI: 1.496–2.653, *p* < 0.001). The optimal response time threshold for survival to hospital discharge was 6.2 min. In the case of OHCA in public areas or with bystander CPR, the threshold was prolonged, and without witness, the optimal response time threshold was shortened.

## 1. Introduction

Out-of-hospital cardiac arrest (OHCA) is defined as the termination of cardiac mechanical activity and subsequent cessation of blood circulation in a patient outside of a hospital [1]. Despite improvements in prehospital management and the use of automated external defibrillators (AEDs), only 10%–20% of the patients who experience OHCA survive to hospital discharge [2, 3].

Many prehospital factors might influence the outcomes of OHCA, such as location of OHCA, witnessed arrest, bystander cardiopulmonary resuscitation (CPR), initial cardiac rhythm, and level of post-resuscitation care [3–6]. The emergency medical service (EMS) response time is defined as the time interval between the call made to the EMS and the arrival of the EMS team at the scene. EMS response time is a key prognostic factor for OHCA, and many studies have shown that short EMS response time is associated with a high probability of survival to hospital discharge and favorable neurologic outcomes [2, 7, 8]. On the other hand, patient-level characteristics, such as sex, age, and comorbidities, might also be prognostic factors of OHCA [9–13]. However, the impact of patient comorbidities on the survival of OHCA patients remains controversial, and Andrew et al. showed that the presence of multiple comorbidities was independently related to a reduced probability of survival to hospital discharge [14]. However, Lai et al. found that cardiac comorbidities might be predictors of improved survival [11]. These results suggest that CPR/AED might be more effective for cardiogenic OHCA than for non-cardiogenic OHCA. Furthermore, the threshold of EMS response time for survival to hospital discharge after OHCA remains unclear. Patient-level differences and conditions of OHCA might influence the response time threshold. For example, Ono et al. found that bystander cardiopulmonary resuscitation (CPR) might prolong the response time threshold from 6.5 min to 7.5 min [7].

Hence, this study aimed to evaluate the EMS response time threshold for survival to hospital discharge for OHCA patients under different conditions, such as patient background and scene of OHCA. To achieve this, we used receiver operating characteristic (ROC) curve analysis and Youden Index.

## 2. Materials and Methods

### 2.1. Study Population

This study was conducted in Kaohsiung, with a population of approximately 2.77 million people, ranked as the third most populated city in Taiwan. The EMS data of OHCA patients were collected from January 2015 to December 2019. The EMS database has been described previously [2]. Briefly, EMS is a single-tiered fire department-based system, maintained by the Taiwanese government, and the data are stored electronically in every province's EMS command center. The EMS database consists of two parts. The first part includes demographic characteristics of the patients, such as age, sex, and comorbidities; details of the scene, such as bystander CPR and location of OHCA; and initial management, as recorded by emergency medical technicians (EMTs). The second part, including the outcome of OHCA patients and disposition, was completed by hospital reviewers. After reviewing the EMS database, we excluded patients aged <18 years [9], those who died due to trauma, cases of drowning, patients with “do not resuscitate” (DNR) orders or with incomplete data, and those transferred to other hospitals after initial resuscitation.

Demographic factors, such as age, sex, and comorbidities, initial management by EMTs, such as the use of defibrillation by automated external defibrillator (AED) or laryngeal mask airway (LMA), and details of the scene, such as bystander CPR and location of OHCA, were recorded in the EMS database. The study was approved by our hospital's institutional review board (number: 202001321B0) and was performed in accordance with the ethical standards set forth in the 1964 Declaration of Helsinki and its later amendments. Formal consent from the patients was not required for this type of study. The primary outcome was survival to hospital discharge.

### 2.2. Statistics

The results of the descriptive analyses of independent variables were presented as mean ± standard deviation (SD). The chi-square test, Mann–Whitney *U* test, and Student's *t*-test were used to analyze independent variables. Logistic regression was used to analyze the statistically significant relationship between prehospital factors, patient comorbidities, and the outcome of OHCA. The odds ratio (OR), 95% confidence interval (CI), and *p* values were also calculated using logistic regression. ROC curve analysis with Youden Index was then used to calculate the optimal cut-off value for EMS response time that predicted survival to discharge under different situations. A *p* value <0.05 was considered statistically significant. All statistical analyses were performed using SPSS version 25.0 (IBM Corp, Armonk, NY, USA).

## 3. Results

A total of 10,933 cases of OHCA during the 5-year study period were recorded in Kaohsiung. We excluded patients aged below 18 years (*n* = 128); cases of burn, trauma, or drowning (*n* = 1,619); patients with DNR orders (*n* = 1,216); patients with missing outcomes or transferred to another hospital (*n* = 564); and patients with incomplete data (*n* = 664). Finally, 6,742 OHCA cases were analyzed in this study.

The demographic characteristics, comorbidities, and prehospital factors are listed in Table 1. A total of 224 OHCA patients survived to hospital discharge. Survival to hospital discharge was associated with young age (*p* < 0.001), male sex (*p*=0.014), presence of witness (*p* < 0.001), public location of cardiac arrest (*p* < 0.001), provision of bystander CPR (*p* < 0.001), bystander airway support (*p*=0.001), EMT-paramedic response ([EMT-P], *p* < 0.001), provision of initial shockable rhythm (*p* < 0.001), defibrillation by AED (*p* < 0.001), and short response time (*p* < 0.001).

Table 2 shows the findings of multivariate logistic regression of OHCA, adjusted for confounding factors of age, male sex, witness, EMS response time, cardiac arrest location (public), bystander CPR, bystander airway maintenance, EMT-P response, initial shockable rhythm, and prehospital defibrillation by AED. After adjusting for confounding factors, age (1 additional year, OR = 0.983, 95% CI: 0.975–0.992, *p* < 0.001), EMS response time (1 minute shorter, OR = 1.217, 95% CI: 1.140–1.299, *p* < 0.001), witness (OR = 3.022, 95% CI: 2.014–4.534, *p* < 0.001), public location (OR = 2.797, 95% CI: 2.062–3.793, *p* < 0.001), bystander CPR (OR = 1.453, 95% CI: 1.071–1.970, *p*=0.016), EMT-P (OR = 1.713, 95% CI: 1.282–2.290, *p* < 0.001), and prehospital defibrillation by AED (OR = 3.984, 95% CI: 2.920–5.435, *p* < 0.001) were statistically and significantly associated with survival to hospital discharge.

Table 3 shows the results of the ROC curve analysis and the optimal response time threshold for predicting survival to hospital discharge. The overall response time threshold was 6.2 min. It was 7.2 min for OHCA occurring in the public area. For patients receiving bystander CPR or those aged <80 years [15], the response time threshold was 6.3 min. For cases without a witness, EMT-P response, and defibrillation with AED and for patients ≥80 years, the time threshold was reduced to 4.2, 5, 5, and 5.1 min, respectively.

**Figure 1:**
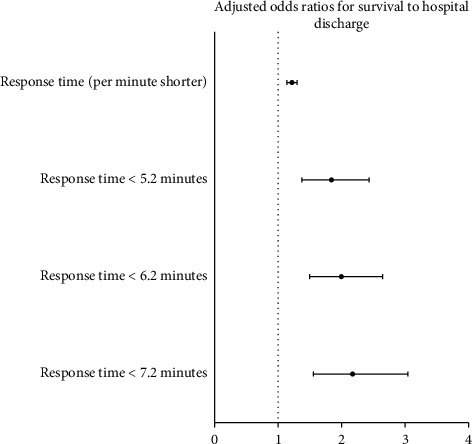
OR and 95% CI for survival to hospital discharge in OHCA patients after adjusting for age, witness presence, public location, bystander CPR, EMT-P attendance, and prehospital defibrillation by AED.

Figure 1 shows the adjusted ORs for survival to hospital discharge of OHCA when the response time cut-off values were set as 5.2 min, 6.2 min, and 7.2 min. The adjusted ORs of per minute shorter response times were 1.217 (95% CI: 1.140–1.299, *p* < 0.001). For response time less than 5.2, 6.2, and 7.2 min, the ORs were 1.832 (95% CI: 1.375–2.440, *p* < 0.001), 1.992 (95% CI: 1.496–2.653), and 2.175 (95% CI: 1.553–3.047), respectively.

## 4. Discussion

In this study, we estimated the EMS response time threshold under different conditions. We found that a short EMS response time was associated with a high rate of survival to hospital discharge after OHCA. The optimal response time threshold for survival to hospital discharge was 6.2 min. In the case of OHCA in public areas or with bystander CPR, the threshold was prolonged to 7.2 min and 6.3 min, respectively; and in the absence of a witness, the threshold was shortened to 4.2 min. Previous studies have focused on the EMS response time threshold for OHCA. Ono et al. collected data from 2,04,277 episodes of bystander-witnessed OHCA and found that the threshold for favorable neurological outcome in OHCA patients was 6.5 min, and the threshold could be prolonged from 1 min to 7.5 min with bystander CPR [7]. Lee et al. demonstrated that the response time thresholds for return of spontaneous circulation (ROSC), survival to discharge, and favorable neurologic outcomes were 11.5, 7.5, and 7.5 min [16]. In the current study, the response time threshold for survival to hospital discharge was 6.2 min. The threshold was shorter in the current study than those reported in previous studies by Ono et al. and Lee et al. One possible explanation for this is the difference in the patient characteristics. Ono et al. only included patients with witnessed OHCA; Lee et al. only included OHCA patients with presumed cardiac etiology by emergency physicians in the ED. We included OHCA patients with or without a witness and with various comorbidities. In our study, the response time threshold for OHCA with a witness was 6.2 min, and the threshold in the study of Ono et al. was 6.5 min. Another possible explanation for the difference is the variation in prehospital management in Taiwan and Japan. In Japan, the ambulance usually has at least one emergency life-saving technician on board, who is allowed to insert an intravenous line. Some of them who are specially trained are allowed to insert tracheal tubes and administer intravenous epinephrine [17]. In Taiwan, EMS agents can be classified as EMT-I, EMT-II, and EMT-P. The differences between EMT-I, EMT-II, and EMT-P lie mainly in the type and duration of the training program and what they are authorized to do. The total training program time to qualify for EMT-I, EMT-II, and EMT-P roles was 40 h, 280 h, and 1280 h, respectively. All EMS agents can perform BLS, LMA insertion, and defibrillation, but only EMT-P agents are authorized to perform advanced life support (ALS) procedures, including intubation, insertion of intravenous lines, and administration of certain medications, such as epinephrine and amiodarone [2]. Ono et al. reported that 29.8% to 33.6% of OHCA patients received an intravenous line; in our study, only 27.0% of the OHCA patients were treated by an EMT-P. This difference might impact the outcome and EMS response time threshold. In fact, the threshold for the group attended by EMT-P (6.2 min) was longer than the threshold for the group attended by other EMTs (5 min). In 2018, the National Development Council of Taiwan set the target response time to 6 min, and the target achievement rate was 90% (https://english.ey.gov.tw/). Mathiesen et al. found that rural regions are associated with prolonged response time and poor outcome [3]; Ho et al. also showed that OHCA occurring at night is associated with prolonged response time [4]. Our results suggest that bystander CPR might extend the threshold of survival to hospital discharge in OHCA patients. Hence, bystander CPR education and earlier recognition of OHCA might help to improve the outcomes of OHCA.

Bystander CPR was found to be associated with survival to hospital discharge and favorable neurological outcomes for OHCA patients [8, 13, 18]. Bystander CPR is associated with a prolonged EMS response time threshold [7]. In our study, bystander CPR prolonged the response time threshold by 0.2 min (6.1 to 6.3 min), but in the study by Ono et al., it prolonged the threshold by 1 min. This variation might be due to the quality of CPR provided by the bystander and the time window between cardiac arrest and CPR initiation. Axelsson et al. collected OHCA data over a 20-year period in Sweden and concluded that OHCA witnessed by an EMS agent had a better prognosis than OHCA not witnessed by an EMS agent [19]. One reason for the good prognosis was the short time window between the collapse and the start of CPR. Sasson et al. reviewed 79 studies and found that 53% (95% CI, 45.0–59.9) of cardiac arrest patients were witnessed by a bystander, and only 32% (95% CI, 26.7–37.8) received bystander CPR [20]. There might be a delay between the collapse and provision of bystander CPR. However, the time window from the onset of cardiac arrest to the start of CPR was not recorded in the present study, and the quality of bystander CPR was difficult to evaluate. Increased awareness of patients and their family members might help them to recognize the warning symptoms of OHCA and initiate timely CPR [19]. In contrast, dispatcher-assisted telephone CPR might shorten the delay time from collapse to CPR initiation, and thus, it is associated with a higher survival rate and better neurological outcomes [21, 22]. In Kaohsiung, the Fire Bureau of Kaohsiung City Government also tried to promote dispatcher-assisted telephone CPR, and the execution rate has also increased in recent years (https://fdkc.kcg.gov.tw/en/). However, some problems exist in telephone CPR, such as incorrect medical condition reporting and quality of CPR performed [23, 24]. Further efforts might focus on dispatcher-assisted telephone CPR, warning symptoms of OHCA recognition, and CPR education.

Many previous studies have shown prehospital factors, such as location of OHCA [3, 25], presence of a witness [20], EMT-P response [2, 26], bystander CPR [18, 27], and prehospital AED use [9, 18, 27]. In the present study, we found that the presence of a witness, public location, bystander CPR, EMT-P response, and defibrillation by AED were independently associated with survival to hospital discharge. Luc et al. examined 6,918 OHCA cases and found that the 30-day survival rate increased from 4.9% to 10.4% when the OHCA event was witnessed and immediate CPR was initiated [27]. Recently, Kern et al. designed a simulation study and found that neighborhood volunteer networks might improve response time [28]. However, there is still no evidence to effectively shorten the time interval from cardiac arrest to CPR initiation in the real world.

Comorbidities can be considered prognostic factors for OHCA, but this point remains controversial. Andrew et al. collected data of 15,953 OHCA patients and found that a high Charlson Comorbidity Index (CCI) was independently associated with low odds of survival to hospital discharge, 1-year functional recovery, and favorable 1-year health-related quality of life [14]. Hirlekar et al. also found that renal disease, diabetes, metastatic carcinoma, and congestive heart failure were independently related to 30-day survival rate [10]. On the other hand, Lai et al. showed that cardiac comorbidities, such as cardiomyopathy and valvular heart disease, were predictors of improved survival in cardiac arrest patients [11]. A review article on 29 observational studies concluded that comorbidities were negatively associated with outcomes in most reported results [29]. Our study did not find a statistically significant difference in comorbidities between the “survival to hospital discharge” and “mortality” groups. One possible reason is that only specific comorbidities were recorded in our study. The lack of a comprehensive view of prearrest comorbidities, such as the CCI, might have influenced the results of this study. Second, prehospital factors, conditions during resuscitation, and post-resuscitation care might counter the impact of prearrest comorbidities. In our study, prehospital factors, such as EMS response time, witnessed OHCA, public location, bystander CPR, EMT-P response, and prehospital defibrillation by AED, may have played a more important role in the prognosis of OHCA than pre-existing morbidities.

There are some limitations to the present study. First, this was a retrospective observational study. Second, the database was restricted to only one city with a single-tiered EMS system, and the results might be different for other cities with different EMS systems. Third, we might have missed OHCA patients who failed to call the EMS and were sent to the hospital by family or health care units. Fourth, our study did not assess the quality of bystander CPR, time of bystander CPR initiation, resuscitation drugs (such as epinephrine, treatment during hospitalization), or dispatcher-assisted CPR.

## 5. Conclusions

We found that a short EMS response time was associated with a high rate of survival to hospital discharge after OHCA. The optimal response time threshold for survival to hospital discharge was 6.2 min. In the case of OHCA in public areas or with bystander CPR, the threshold was prolonged to 7.2 min and 6.3 min, respectively; and in the absence of a witness, the threshold was shortened to 4.2 min.

## Figures and Tables

**Table 1:** Demographic characteristics, comorbidities, and prehospital factors of 6742 medical out-of-hospital cardiac arrest patients.

Characteristics of medical out-of-hospital cardiac arrest (OHCA) patients	Survived to hospital discharge *n* = 224 (3.3%)	Did not survive to hospital discharge *n* = 6518 (96.7%)	*p*
Age (years)	60.9 ± 14.4	68.3 ± 16.1	<0.001
Male sex	162 (72.3%)	4190 (64.3%)	0.014
EMS response time (min)	5.8 ± 2.2	7.0 ± 3.2	<0.001
Witnessed arrests	192 (85.7%)	3872 (59.4%)	<0.001
Cardiac arrest location (public)	101 (45.1%)	1064 (16.3%)	<0.001
Bystander CPR	117 (52.2%)	2536 (38.9%)	<0.001
Bystander keep airway	27 (12.1%)	418 (6.4%)	0.001
Attended by EMS-paramedic	88 (39.3%)	1730 (26.5%)	<0.001
Initial shockable rhythm	34 (15.2%)	191 (2.9%)	<0.001
Defibrillation by AED	113 (50.4%)	875 (13.4%)	<0.001
Hypertension	89 (39.7%)	2284 (35.0%)	0.254
Diabetes	59 (26.3%)	1637 (25.1%)	0.807
Old stroke	19 (8.5%)	478 (7.3%)	0.617
Malignancy	15 (6.7%)	522 (8.0%)	0.41
Liver disease	10 (4.5%)	207 (3.2%)	0.326
Respiratory disease	12 (5.4%)	265 (4.1%)	0.401
Renal disease	25 (11.2%)	579 (8.9%)	0.3

**Table 2:** Adjusted odds ratios for survival to hospital discharge.

Adjusted odds ratios for outcome	Survival to hospital discharge		
Variables	OR	95% CI	*p*
Response time (one minute shorter)	1.217	1.140–1.299	<0.001
Age (one additional year)	0.983	0.975–0.992	<0.001
Male sex	0.901	0.653–1.245	0.529
Witness	3.022	2.014–4.534	<0.001
Cardiac arrest location (public)	2.797	2.062–3.793	<0.001
Bystander CPR	1.453	1.970–1.071	0.016
Bystander keep airway	1.022	0.638–1.636	0.928
Attended by EMT-Paramedic	1.713	1.282–2.290	<0.001
Initial shockable rhythm	1.542	0.986–2.411	0.057
Defibrillation by AED	3.984	2.920–5.435	<0.001

**Table 3:** Receiver operating characteristic (ROC) curve analysis of the optimal response time threshold for predicting survival to hospital discharge.

Situation		Survival to hospital discharge				
		Threshold (minutes)	AUC	Lower	Upper	*p*
Overall		6.2	0.618	0.582	0.654	<0.001
Witnesses	With	6.2	0.609	0.570	0.647	<0.001
	Without	4.2	0.643	0.544	0.743	<0.001
Cardiac arrest location	Public	7.2	0.628	0.576	0.681	<0.001
	Residence	6.1	0.611	0.563	0.66	<0.001
Bystander CPR	With	6.3	0.588	0.537	0.64	0.001
	Without	6.1	0.655	0.606	0.703	<0.001
Attended by EMS-paramedic	With	6.2	0.635	0.581	0.689	<0.001
	Without	5.0	0.605	0.558	0.653	<0.001
Defibrillation by AED	With	6.2	0.619	0.568	0.669	<0.001
	Without	5.0	0.632	0.572	0.675	<0.001
Age	<80 years	6.3	0.627	0.589	0.665	<0.001
	≥80 years	5.1	0.588	0.524	0.651	0.007

## Data Availability

The datasets used and analyzed during the current study are available from the corresponding author on reasonable request.
